# A new clamp for bilamellar tarsal rotation for trachomatous trichiasis

**Published:** 2009-03

**Authors:** Keith Waddell

**Affiliations:** Mobile Ophthalmologist, Ruharo Health Centre, PO Box 14, Mbarara, Uganda. Email: Ruharo@bushnet.net

**Figure:**
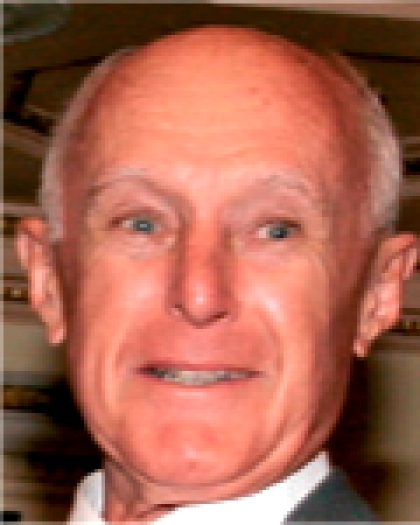


Surgery for trichiasis is an important part of the SAFE strategy for avoiding blindness and pain from trachoma. It often has to be performed by non-specialist workers in remote places. The World Health Organization (WHO) is rightly promoting bilamellar rotation as a standard operation,^1^ but its method presents difficulties even for trained surgeons.^2^ In the technique described by WHO, the lid is held by two artery forceps; however, it is difficult to make accurate cuts this way. Also, after the forceps are taken off, stitches then have to be put in a floppy, bleeding lid. A clamp is needed to stabilise the lid and prevent bleeding, but none of the available ones are suitable.

**Figure 1:**
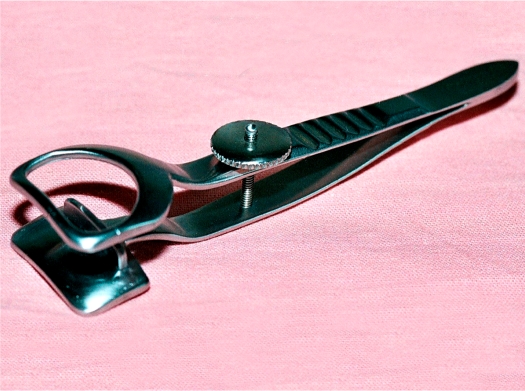
The new lid rotation clamp

**Figure 2:**
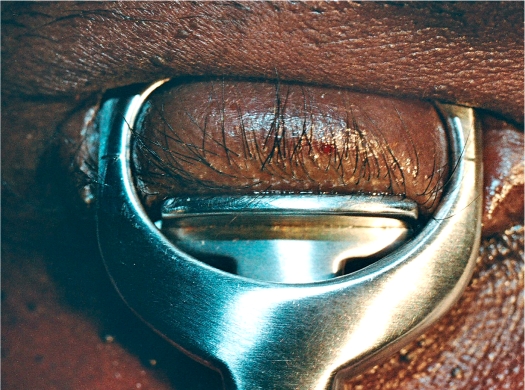
The clamp in place; a mark at 3 mm shows where to cut

I have prepared a new design of clamp, which has worked well in the field in Uganda and Sudan, including when it was used by nurse surgeons. The clamp is shaped to hold the two layers correctly together, with the lid margin lying against a shelf (Figure 1). When inserted correctly, the lid is stable and bloodless. There is a mark at 3 mm from the margin to show exactly where to cut (Figure 2). The cut is made through both layers together, down to the plate guarding the eye.

Keith Waddell

Keith Waddell

**Figure 3:**
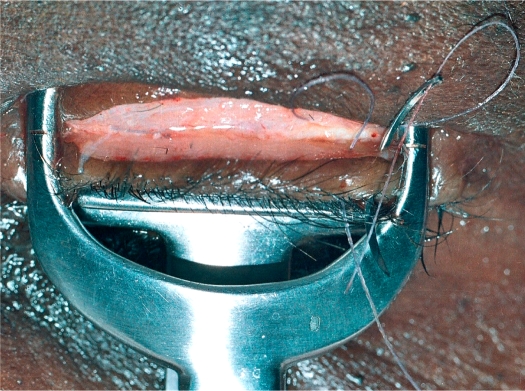
Inserting the first stitch after the cut has been completed

Whilst the lid is still held in the clamp, the stitches can be put in easily and accurately. Figure 3 shows the cut completed and the first stitch being inserted. Stitches start through the skin and muscle of the lid edge, then through the upper tarsal plate partial thickness side to side, and finally back beside the first bite. The stitches at the ends of the incision are tied firmly whilst still in the clamp. The central stitch is left loose until the clamp is taken off, so it can be adjusted accurately.

Keith Waddell

**Figure 4:**
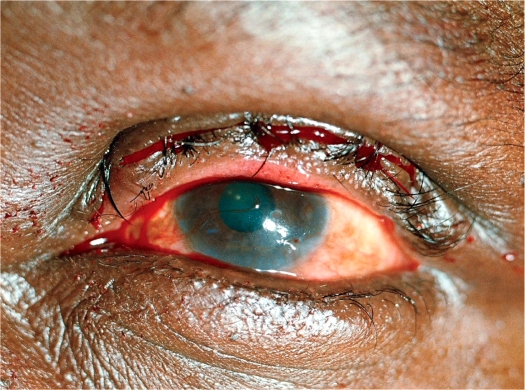
The final result

Only one short piece of stitch and one needle are used for all stitches, as opposed to three double-armed in the WHO method, so it is economic to use absorbable suture and the patient need not return for stitch removal. The end result is a neat operation, likely to be successful (Figure 4).

Keith Waddell

For very severe cases with total in-turning of all lashes, however, I find this operation is not successful: a Trabut procedure is needed, as other surgeons have also found.^3^

The clamp is available from: Collton Hailsham Ltd, Unit 1B, Hankham Hall Cottage, Hankham Hall Road, Westham, Pevensey, West Sussex, BN24 5AH, UK. Tel/Fax: +44 1323 743629. Email: colltonhailsham@btconnect.com The clamp comes with a leaflet describing its correct use. A CD-ROM of the procedure will be available soon. We have found the clamp fits most eyes (even children's), but smaller and larger models are available for the few patients with very contracted or large conjunctival sacs.

**Declaration of interest:** the author has no commercial interest in the clamp. Suggestions for improvement are welcome.
